# Can the allocation of primary health care system resources affect efficiency? A spatial Dubin model study in China

**DOI:** 10.1186/s12875-024-02290-y

**Published:** 2024-02-21

**Authors:** Xinyue Sun, Bo Lv, Xiaoyi Gao, Kai Meng

**Affiliations:** 1 School of Public Health, Capital Medical University, No.10 Xitoutiao, Youanmenwai Street, Fengtai District, Beijing, 100069 China; 2 School of Medical Humanities, Capital Medical University, No.10 Xitoutiao, Youanmenwai Street, Fengtai District, Beijing, 100069 China; 3 Beijing Tiantan Hospital, Capital Medical University, No.119 South of the Fourth Ring Road, Fengtai District, Beijing, 100069 China

**Keywords:** Primary health care, Health resources allocation, Efficiency, Spatial Dubin model

## Abstract

**Background:**

The primary health care (PHC) system plays an important role in China’s health care system, but there are challenges such as irrational allocation of health resources and inefficient operation, which need to be improved. The purpose of this study was to explore the impact of resource allocation on the efficiency of the PHC system in China.

**Methods:**

The data in 31 provinces were collected from the China Statistical Yearbook 2017–2021 and the China Health Statistical Yearbook 2017–2021. The comprehensive health resource density index (CHRDI) was constructed based on the entropy method and the health resource density index (HRDI), which was used to analyze the allocation of primary health resources in each province. The adjusted efficiency of the PHC system in each province was calculated by the bootstrap data envelopment analysis (DEA). Finally, the spatial Dubin model was used to explore the effect of the CHRDI on efficiency.

**Results:**

From 2016 to 2020, the allocation of primary health resources in 31 provinces showed an increasing trend, and the average efficiency after correction showed a decreasing state year by year. The spatial direct effect and spatial spillover effect coefficients of CHRDI were 0.820 and 1.471, which positively affect the efficiency. Per capita Gross Domestic Product (GDP), urbanization rate, and the proportion of the elderly were the factors affecting the efficiency of the PHC system.

**Conclusions:**

The allocation of primary health resources in all provinces in China has improved each year, but there are still great differences, and efficiency must be further improved. Pay attention to the spatial spillover effect of the level of resource allocation and formulate differentiated measures for different regions. Attention should also be paid to the impact of population aging and economic development on the utilization of primary health resources by increasing health needs and choices.

**Supplementary Information:**

The online version contains supplementary material available at 10.1186/s12875-024-02290-y.

## Background

Universal access to quality primary health care (PHC) is a common goal pursued by countries around the world. PHC system is a key component of all health systems in the world and plays an important role in ensuring access to basic health services [1]. Reform of the PHC system is necessary to achieve universal health coverage [2, 3]. China’s PHC institutions are the main places that provide PHC services, with functions that include diagnosis, referral, and health management [4]. Rational allocation of primary health resources has always been the goal pursued by health administrators [5]. Since 2009, China has been actively promoting the deepening of the medical and health system reform to meet the residents’ demand for health services, which depends on the rational allocation of primary health resources as well as the improvement of service efficiency and service quality [6]. Through the government’s relentless efforts, the frequency of residents’ utilization of PHC institutions has increased [7]. At the same time, the role of the PHC system is increasingly prominent due to the accelerated aging of China and the increased prevalence of chronic diseases.

Improving the efficiency of the PHC system is an important path to achieve the goal of “strengthening the grassroots” in new healthcare reform. Since 2009, China has introduced a series of policies in terms of financial support, operation and maintenance, service provision, and talent development, and has made large-scale investments in the PHC system [8]. Evaluating the efficiency of the PHC system and exploring the factors influencing the efficiency has been a hot topic. Scholars have used different Data Envelopment Analysis models to analyze the PHC system after the new healthcare reform, their results showed that the efficiency of the PHC system was still at a low level [9, 10], because the function of the PHC system has been marginalized, and it takes a longer time to rebuild the trust of patients. Cheng et al. evaluated the productivity of rural township hospitals in China and found that productivity first increased from 2008 to 2012, and then decreased after 2012 [11], Su et al. also confirmed this phenomenon [12]. The new healthcare reform’s investment in primary health resources has improved the situation, but there seemed to be no sustained improvement. Firstly, the PHC system’s efficiency is affected by a variety of factors, the financial subsidy method, population size, aging level, resident income level, urbanization rate, and population health level are the factors that affect the efficiency of the PHC system [8, 13]. Secondly, the resources invested by the government can only be improved in the long run if they are rationally allocated.

Health resources are scarce in all countries, and the rational allocation of health resources is closely related to the quality of medical services [14]. Rational allocation of primary health resources can ensure equity in the supply of PHC services, promote sustainable development, and continuously improve the health level of the population [15]. Since the new healthcare reform initiative, China has introduced several policies that promote the rational allocation of primary health resources, but at present, there are still vast differences in how the allocation of primary health resources is disseminated among provinces, which hinders the development of PHC services and cannot fully meet the basic medical service needs of residents. Further measures are needed to improve the rationality of their allocation [16–18]. The impact of rational allocation of health resources on improving efficiency is mostly qualitative studies. Qin et al. believed that the continuous improvement of the rational allocation of primary health resources in China has a positive impact on the efficiency of the PHC system [19]. Su et al. suggested synergy between comprehensive healthcare resource allocation and efficiency in hospital systems but did not explore the quantitative relationship between them [12]. There may be a complex relationship between resource allocation and efficiency in the PHC system [20], but there is a lack of relevant quantitative research. Therefore, the purpose of this study is to assess the level of resource allocation in the PHC system and its impact on efficiency.

## Materials and methods

### Data sources

In this study, the PHC system includes community health centers (stations), township (street) health centers, village health offices, outpatient clinics, and clinics (medical offices). There are 34 provinces (municipalities directly under the Central Government and autonomous regions) in China. Due to the availability of data, Hong Kong, Macao, and Taiwan in China were not included in this study. A total of 31 provinces (municipalities directly under the Central Government and autonomous regions) were selected for the number of primary health care institutions, beds, health technicians, non-health technicians, outpatient visits, inpatient visits, and home health services, which were used to analyze the resources allocation and efficiency of the PHC system. The data were derived from the China Statistical Yearbook (2017–2021). Population density, per capita GDP, urbanization rate, the proportion of the elderly, the proportion of government health expenditure in fiscal expenditure, the proportion of hospitals in health institutions, and the proportion of service volume of PHC system in health institutions were derived from the China Statistical Yearbook (2017–2021). No human data were taken in this study, and all data on the PHC system were open to the public.

### Comprehensive health resource density index (CHRDI)

The CHRDI was constructed based on the entropy method and the health resource density index (HRDI). First, the entropy method was used to assign weights to different PHC system resource indicators to obtain the comprehensive health resource indicator, and then the CHRDI of the comprehensive health resource indicator was calculated.

### The entropy method

The entropy method is one of the methods used to objectively assign weights to indicators [21]. Health resources mainly include human, material, and financial resources. This study assigned weights to the number of PHC institutions, the number of health technicians, the number of beds, and the financial subsidies in 31 provinces [12]. The comprehensive health resource indicator was formed by calculating the following steps:

1. Standardization of indicators:
  When ${X}_{ij}$ is a positive indicator: ${X}_{ij}={X}_{ij}{-X}_{min(j)}/{X}_{max(j)}-{X}_{min(j)}$
  When ${X}_{ij}$ is a negative indicator: ${X}_{ij}={X}_{max(j)}-{X}_{ij}/{X}_{max(j)}-{X}_{min(j)}$
2. Measure the value of the weight of the $ith$ indicator under the $jth$ indicator ${P}_{ij}$:
  $${P}_{ij}={X}_{ij}^{\prime}/\sum\limits_{i=1}^{m}{X}_{ij}^{\prime}$$
3. Calculate the entropy value of the jth indicator ${e}_{j}$:
  $${e}_{j}=-\frac{1}{lnm}\sum_{i=1}^{m}{P}_{ij}ln{P}_{ij}$$
4. The difference coefficient ${g}_{i}$ of the $ith$ indicator:
  $${g}_{i}=1-{e}_{i}$$
5. The weight of the jth indicator ${W}_{j}$:
  $${W}_{j}={g}_{i}/\sum_{i=1}^{m}{g}_{i}$$

According to the indicator weight constructed by the entropy method, the comprehensive health resource indicator ${u}_{i}$ was calculated, which combined the number of PHC institutions, the number of health technicians, the number of beds, and the financial subsidies. The formula was as follows:

$${u}_{i}=\sum_{j=1}^{m}{W}_{j}{X}_{ij}$$

### Health resource density index (HRDI)

The HRDI is a comprehensive measure of the allocation of health resources from both population and geographic dimensions, solving the problem that the analysis from both population and geographic dimensions may vary more [22, 23]. In this study, the comprehensive health resource density index (CHRDI) was calculated with the following formula:

$${\text{CHRDI}}=\sqrt{\frac{\mathrm{Comprehensive\ health\ resources\ index}}{\mathrm{ thousands\ of\ people}}\times \frac{\mathrm{Comprehensive\ health\ resources\ index}}{\mathrm{Square\ kilometer}}}$$

### The bootstrap DEA

The traditional input-oriented invariant returns to scale DEA model obtains the technical efficiency (TE) scores of PHC systems in each province and city by solving the following linear programming problems.

$$min\theta$$

$$\theta {x}_{i}-X\lambda \ge 0$$

$$Y\lambda \ge {y}_{i}$$

$$\lambda \ge 0$$

where $\theta$ is the TE score of the PHC system in province i, $X$ and $Y$ are the input and output variable matrices, ${x}_{i}$ and ${y}_{i}$ are the input and output vectors of the PHC system in province i, and $\lambda$ is the weight vector. The value of $\theta$ is between 0 and 1, with a score of 1 indicating an effective frontier in terms of technical efficiency.

The bootstrap method, first proposed by Efron [24], is an unbiased estimation method based on iterative sampling. This study used the bootstrap DEA developed by Simar for bias correction. The total was inferred by sampling existing samples with put-back, which solved the shortcomings of traditional DEA that did not consider random errors, thus improving the accuracy of efficiency measurement [25–27]. This study applied the bootstrap method and selected an input-oriented scale variable model to estimate the unbiased efficiency score by sampling 2,000 times with retracting.

The input and output indicators for this study were selected by reviewing the literature and based on the availability of data in the National Health Statistics Information Reporting System [28, 29]. The input indicators mainly considered capital and human resources, and the number of institutions and beds represent capital. In terms of human resources, considering that PHC institutions are smaller than hospitals and the number of health workers is small, non-health workers are equally important. Therefore, the human resources were divided into health workers and non-health workers. The health workers included health technicians and rural doctors, and the non-health workers included administrative staff and other non-clinical staff. The output indicators were mainly healthcare service utilization indicators. Considering that the current PHC system suffers from irrational allocation, we paid more attention to how to obtain a larger healthcare service utilization through fewer inputs. Healthcare utilization to improve the health level of residents is a complex process that requires different healthcare systems to work together, and it may be difficult to populate multi-stage data for DEA when a complex production process is involved [30], so we did not collect output indicators for health levels. The output indicators included the number of consultations, hospital admissions, and unique family health service visits [31].

We constructed the comprehensive distribution plot of efficiency and CHRDI with CHRDI as the horizontal coordinate and efficiency as the vertical coordinate, with the average of the two as the intersection of the horizontal and vertical coordinates. The first quadrant indicates high allocation of health resources and high efficiency; the second quadrant indicates low allocation of health resources and high efficiency; the third quadrant indicates low allocation of health resources and low efficiency; and the fourth quadrant indicates high allocation of health resources and low efficiency.

### The spatial econometric model

The spatial autocorrelation and the spatial Dubin model were used to explore the impact of primary resource allocation on efficiency. First, Moran’s index in the spatial autocorrelation model was used to assess the degree of spatial autocorrelation in the dependent variable (efficiency). When spatial autocorrelation was present in data, the spatial Dubin model was constructed to inform about the level of influence of the independent variable (CHRDI) or the underlying spatial dependence structure.

### The Moran’s index

Before building a spatial econometric model, it is necessary to analyze the spatial autocorrelation of the dependent variable to reflect the degree of interdependence of factor attributes on spatial location. The Moran’s index is a measure of the degree of spatial agglomeration. The global Moran’s index (Moran’s I) can test the spatial dependence of the overall regional efficiency with the following equation:

$$\mathrm{global\ Moran^{\prime}}\mathrm{s\ I}=\frac{{\sum }_{{\text{i}}=1}^{{\text{N}}}\sum_{{\text{y}}=1}^{{\text{N}}}{{\text{W}}}_{{\text{i}},{\text{j}}}({{\text{TE}}}_{{\text{i}},{\text{t}}}-\overline{{{\text{TE}} }_{{\text{t}}}})({{\text{TE}}}_{{\text{j}},{\text{t}}}-\overline{{{\text{TE}} }_{{\text{t}}}})}{[\frac{1}{{\text{N}}}{\sum }_{{\text{i}}=1}^{{\text{N}}}{({{\text{TE}}}_{{\text{i}},{\text{t}}}-\overline{{{\text{TE}} }_{{\text{t}}}})}^{2}]\ {\sum }_{{\text{i}}=1}^{{\text{N}}}{\sum }_{{\text{j}}=1}^{{\text{N}}}{{\text{w}}}_{{\text{i}},{\text{j}}}}$$

${\text{N}}$ represents 31 provinces and cities, and ${{\text{W}}}_{{\text{i}},{\text{j}}}$ represents the spatial weight matrix between provinces i and j. In this study, the spatial inverse distance matrix most consistent with the cognition of spatial relationship was selected. The greater the distance between regions, the smaller the weight, and the closer the distance, the greater the influence weight. The global Moran’s I range from [− 1,1], and $\overline{TE }$ is the average value of ${\text{TE}}$; a value greater than 0 indicates a positive spatial dependence of the ${\text{TE}}$, while a value less than 0 indicates a negative spatial autocorrelation.

The global Moran’s I can reflect the overall spatial correlation of TE but may ignore some local features. The local Moran’s I can examine the degree of clustering or dispersion in the local area, which was calculated as:

$$\mathrm{local\ Moran^{\prime}}\mathrm{s\ I}=\frac{{\text{N}}({{\text{TE}}}_{{\text{i}},{\text{t}}}-\overline{{{\text{TE}} }_{{\text{t}}}}){\sum }_{{\text{j}}=1}^{{\text{N}}}{{\text{W}}}_{{\text{i}},{\text{j}}}({{\text{TE}}}_{{\text{j}},{\text{t}}}-\overline{{{\text{TE}} }_{{\text{t}}}})}{{\sum }_{{\text{i}}=1}^{{\text{N}}}{({{\text{TE}}}_{{\text{i}},{\text{t}}}-\overline{{{\text{TE}} }_{{\text{t}}}})}^{2}]}$$

We classified the 31 provinces into four clusters based on the results of the local Moran’s I: The high-high (HH) cluster represented that a province with a high TE value was close to other provinces with a high TE value. The low–high (LH) cluster represented the province with a low TE value but was surrounded by provinces with a high TE value. The same principle could be obtained for the low-low (LL) cluster and the high-low (HL) cluster.

### The spatial Dubin model

When efficiency had significant spatial correlation characteristics, the spatial econometric model needed to be introduced at this time to explore the factors influencing efficiency [32]. There are three classical spatial econometric models: the spatial Lag model (SLM), the spatial Error model (SEM), and the spatial Dubin model (SDM). Compared with the spatial Lag model and the spatial Error model, the spatial Dubin model is more comprehensive. The likelihood ratio (LR) test was also used to reconfirm that the spatial Dubin model would not degenerate into a spatial lag model or a spatial error model. The Hausman test showed that the fixed effects model rather than the random effects model was applied in this study. All tests passed the 1% significance level test, and the dynamic fixed-effects SDM was finally selected to explore the effect of health resource allocation on efficiency. The formula is as follows:

$${\text{Y}}=\mathrm{\rho WY}+\mathrm{\beta X}+\mathrm{\theta WX}+\upvarepsilon$$

${\text{Y}}$ is the explained variable; ${\text{X}}$ is the explanatory variable; $\uprho$ is the spatial autoregressive coefficient; $\upbeta$ represents the regression spatial coefficient; ${\text{W}}$ represents the space weight matrix; $\upvarepsilon$ represents the random error term; $\uptheta$ represents the spatial autoregressive coefficient of the explanatory variable. In this study, the following control variables were selected by reviewing the literature. The control variables were divided into external environment variables and health system variables. population density, per capita GDP, urbanization rate, the proportion of the elderly, and the proportion of government health expenditure in fiscal expenditure were selected as external environment control variables, reflecting economic development level, urban construction level, population distribution, population structure, and government financial input. We also paid attention to the impact that the distribution of health resources and health service utilization in the health system might have on the efficiency of the PHC system, so we also included the proportion of hospitals in health institutions and the proportion of service volume of PHC system in health institutions [33–38] (Table 1).

**Table 1:** Variable selection of the spatial Dubin model

	Index name	Calculation method	Predicted impact
	Comprehensive health resource density index		positive
External environment variables	Population density	Resident population/total area of the region	positive
	Per capita GDP	Gross regional product/resident population	negative
	Urbanization rate	Regional urban population/regional permanent population	positive
	Proportion of the elderly	The proportion of people over 65 in the total population	uncertain
	Proportion of government health expenditure in fiscal expenditure	Local financial health care Expenditure/regional GDP	positive
Health system variables	Proportion of hospitals in health institutions	Number of hospitals/number of health institutions	negative
	Proportion of service volume of PHC system in health institutions	Number of PHC system treatment volume/number of treatment volume institutions treatment volume	positive

### Data analysis

Microsoft Excel 2019 was used to calculate the comprehensive health resource density index. Stata 17.0 was used in plotting the distribution of average efficiency, MaxDEA 7 Ultra for Bootstrap-dea, and Stata 17.0 for spatial econometric models.

## Results

### Status of primary comprehensive health resources allocation

The four indicators of the number of PHC institutions, the number of health technicians, the number of beds, and the financial subsidies were assigned weights by the entropy method. The weights of the four indicators did not change significantly from year to year, and the weights of the number of institutions and the number of beds were slightly higher. The comprehensive allocation of primary health resources in all provinces showed an upward trend. The CHRDI in almost all provinces showed an increasing trend, indicating that the allocation of primary health resources in China was gradually improving and the allocation of resources was more rational. The CHRDI of Shanghai, Beijing, and Tianjin were significantly higher than other provinces, indicating that these provinces had a relatively high allocation of PHC system resources based on a combination of population size and geographic area (Table 2).

**Table 2:** Comprehensive health resource density index, 2016–2020

	2016	2017	2018	2019	2020
Beijing	2.914	2.821	3.275	3.495	3.628
Tianjin	1.905	1.986	2.092	2.143	2.275
Hebei	1.087	0.970	1.226	1.247	1.326
Shanxi	0.961	0.858	1.015	1.037	1.087
Inner Mongolia	0.300	0.280	0.327	0.331	0.351
Liaoning	0.993	0.913	1.060	1.045	1.092
Jilin	0.693	0.638	0.761	0.758	0.879
Heilongjiang	0.490	0.451	0.516	0.535	0.569
Shanghai	3.720	3.624	4.062	4.208	4.454
Jiangsu	1.530	1.369	1.731	1.820	1.967
Zhejiang	1.379	1.382	1.507	1.575	1.701
Anhui	0.940	0.814	1.046	1.095	1.291
Fujian	0.926	0.844	1.002	1.034	1.129
Jiangxi	0.853	0.722	0.932	0.965	1.048
Shandong	1.541	1.361	1.692	1.726	1.834
Henan	1.310	1.116	1.440	1.467	1.628
Hubei	1.125	0.920	1.182	1.177	1.306
Hunan	1.117	0.895	1.188	1.284	1.326
Guangdong	1.205	1.146	1.332	1.379	1.484
Guangxi	0.837	0.705	0.892	0.924	1.029
Hainan	0.877	0.884	0.952	0.982	1.170
Chongqing	1.169	0.949	1.284	1.340	1.445
Sichuan	0.843	0.689	0.918	0.949	1.015
Guizhou	0.795	0.704	0.897	0.944	1.039
Yunnan	0.566	0.518	0.659	0.707	0.781
Tibet	0.099	0.109	0.112	0.118	0.128
Shaanxi	0.917	0.861	0.998	1.038	1.090
Gansu	0.426	0.387	0.473	0.506	0.531
Qinghai	0.177	0.192	0.212	0.214	0.226
Ningxia	0.551	0.622	0.634	0.648	0.699
Xinjiang	0.257	0.220	0.260	0.272	0.285

### Status of input and output indicators of the PHC system

From 2016–2020, the number of PHC institutions, beds, health technicians, and non-health technicians in China increased, with the number of non-health technicians growing the fastest. In terms of output indicators, both the number of patients and the number of hospital admissions increased first and then decreased. In 2020, affected by the COVID-19 epidemic, the number of family health service visits decreased, but the overall growth trend was obvious (Table 3).

**Table 3:** Status of input and output indicators of PHC system, 2016–2020

	Inputs				Outputs		
	Number of institutions	Number of beds	Number of health workers	Number of non-health workers	Number of consultations	Number of hospital admissions	Family health service visit
2016	29,888	46,514	108,218	10,574	14,086	134	480,950
2017	30,098	49,307	112,058	11,369	14,287	144	648,917
2018	30,440	51,083	115,809	12,086	14,214	141	686,962
2019	30,787	52,617	121,397	12,815	14,616	139	825,223
2020	31,291	53,206	126,434	13,557	13,278	120	856,881

### Technical efficiency of the PHC system

**Fig. 1:**
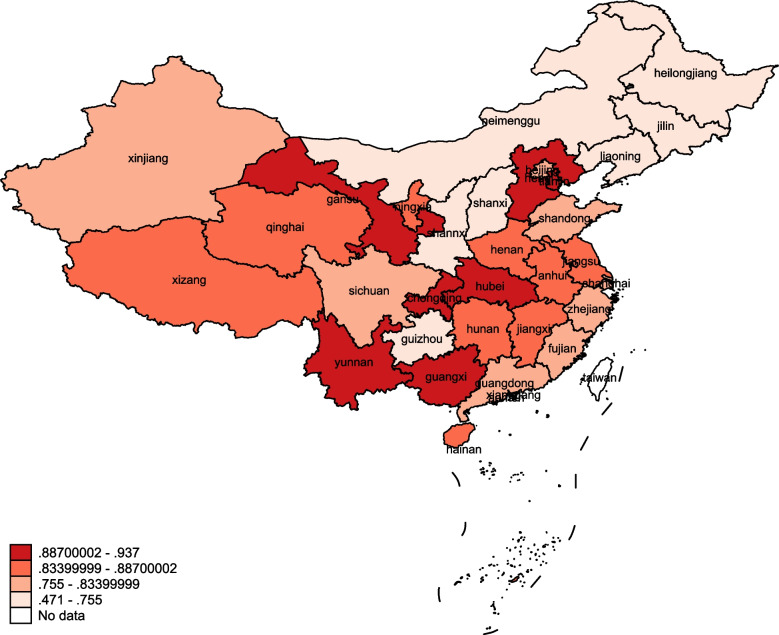
Average technical efficiency of PHC systems in 31 provinces, 2016–2020

After the 2000 sampling of efficiency was put back through Bootstrap-DEA, the efficiency values after deviation correction were lower than those before deviation correction. After deviation correction, the average efficiency values from 2016 to 2020 showed a downward trend year by year, with a significant decrease in 2019 and 2020. No regional efficiency score reached 1. In terms of geographical distribution, the province with the highest 5-year average efficiency was Guangxi (0.937), while Jilin Province had the lowest efficiency mean (0.471) (Table 4). In terms of overall geographic distribution, the technical efficiency of China’s PHC system was characterized by “better in the center and worse in the east and west” (Fig. 1).

**Table 4:** Technical efficiency of PHC system based on Bootstrap-DEA, 2016–2020

Region	2016		2017		2018		2019		2020		Mean
	Traditional	Correction	Traditional	Correction	Traditional	Correction	Traditional	Correction	Traditional	Correction	
Beijing	1.000	0.826	1.000	0.840	1.000	0.825	1.000	0.766	1.000	0.750	0.801
Tianjin	1.000	0.958	1.000	0.933	1.000	0.895	1.000	0.840	1.000	0.909	0.907
Hebei	1.000	0.920	1.000	0.938	0.992	0.962	0.941	0.886	0.856	0.808	0.903
Shanxi	0.545	0.531	0.534	0.515	0.583	0.561	0.499	0.464	0.493	0.460	0.506
Inner Mongolia	0.594	0.577	0.594	0.577	0.610	0.586	0.500	0.465	0.499	0.474	0.536
Liaoning	0.641	0.627	0.655	0.638	0.668	0.653	0.637	0.609	0.560	0.543	0.614
Jilin	0.489	0.475	0.526	0.514	0.515	0.501	0.491	0.465	0.424	0.400	0.471
Heilongjiang	0.735	0.717	0.714	0.698	0.557	0.540	0.482	0.456	0.416	0.397	0.562
Shanghai	1.000	0.837	1.000	0.836	1.000	0.826	1.000	0.765	1.000	0.755	0.804
Jiangsu	1.000	0.879	1.000	0.834	1.000	0.848	1.000	0.815	1.000	0.829	0.841
Zhejiang	1.000	0.851	1.000	0.828	1.000	0.839	1.000	0.773	1.000	0.739	0.806
Anhui	1.000	0.945	1.000	0.901	1.000	0.889	1.000	0.776	1.000	0.759	0.854
Fujian	0.770	0.750	0.741	0.719	0.766	0.744	0.785	0.742	0.861	0.818	0.755
Jiangxi	1.000	0.907	1.000	0.872	1.000	0.878	1.000	0.853	1.000	0.896	0.881
Shandong	1.000	0.841	1.000	0.842	1.000	0.834	1.000	0.872	1.000	0.749	0.828
Henan	1.000	0.933	1.000	0.917	1.000	0.939	0.966	0.888	1.000	0.756	0.887
Hubei	1.000	0.870	1.000	0.908	1.000	0.914	1.000	0.893	1.000	0.941	0.905
Hunan	1.000	0.887	1.000	0.907	1.000	0.876	1.000	0.818	1.000	0.824	0.862
Guangdong	1.000	0.836	1.000	0.829	1.000	0.834	1.000	0.777	1.000	0.743	0.804
Guangxi	1.000	0.942	0.987	0.959	0.993	0.968	1.000	0.902	1.000	0.912	0.937
Hainan	0.936	0.899	0.906	0.869	0.904	0.864	0.901	0.834	0.929	0.879	0.869
Chongqing	1.000	0.904	1.000	0.890	1.000	0.897	1.000	0.869	1.000	0.878	0.888
Sichuan	1.000	0.825	1.000	0.827	1.000	0.842	1.000	0.782	1.000	0.748	0.805
Guizhou	0.761	0.742	0.744	0.725	0.813	0.794	0.835	0.791	0.729	0.699	0.750
Yunnan	1.000	0.939	0.954	0.921	0.969	0.937	1.000	0.924	1.000	0.938	0.932
Tibet	1.000	0.895	1.000	0.905	1.000	0.853	1.000	0.784	1.000	0.810	0.849
Shaanxi	0.727	0.704	0.774	0.750	0.771	0.745	0.748	0.700	0.654	0.627	0.705
Gansu	1.000	0.940	1.000	0.922	1.000	0.919	0.987	0.914	1.000	0.933	0.926
Qinghai	1.000	0.833	1.000	0.847	1.000	0.842	1.000	0.848	1.000	0.903	0.855
Ningxia	1.000	0.848	1.000	0.851	1.000	0.861	1.000	0.823	1.000	0.789	0.834
Xinjiang	0.930	0.905	0.852	0.822	0.770	0.744	0.799	0.750	0.729	0.702	0.785
Mean	0.891	0.813	0.887	0.807	0.884	0.802	0.867	0.755	0.847	0.735

### Relationship between resource allocation and efficiency of the PHC system

**Fig. 2:**
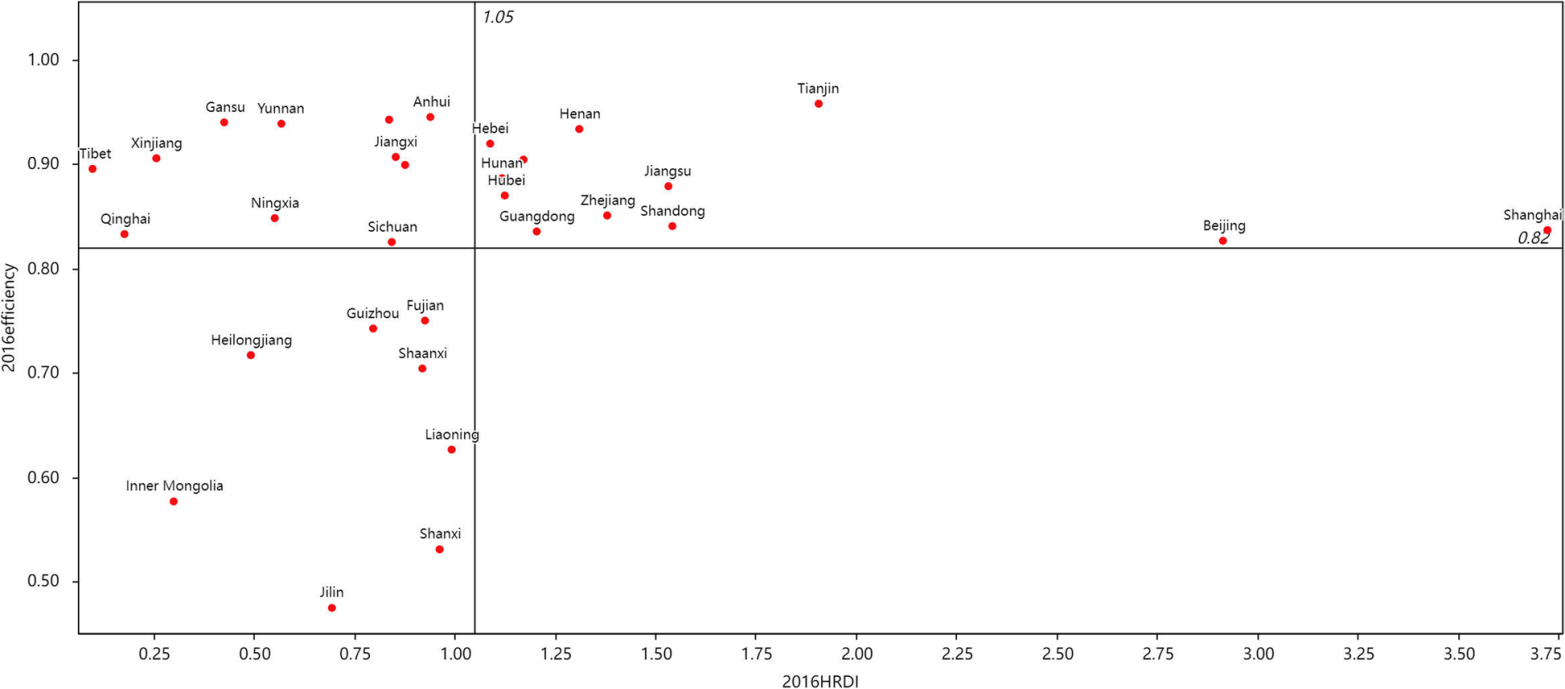
The comprehensive distribution plot of efficiency and CHRDI in 2016

**Fig. 3:**
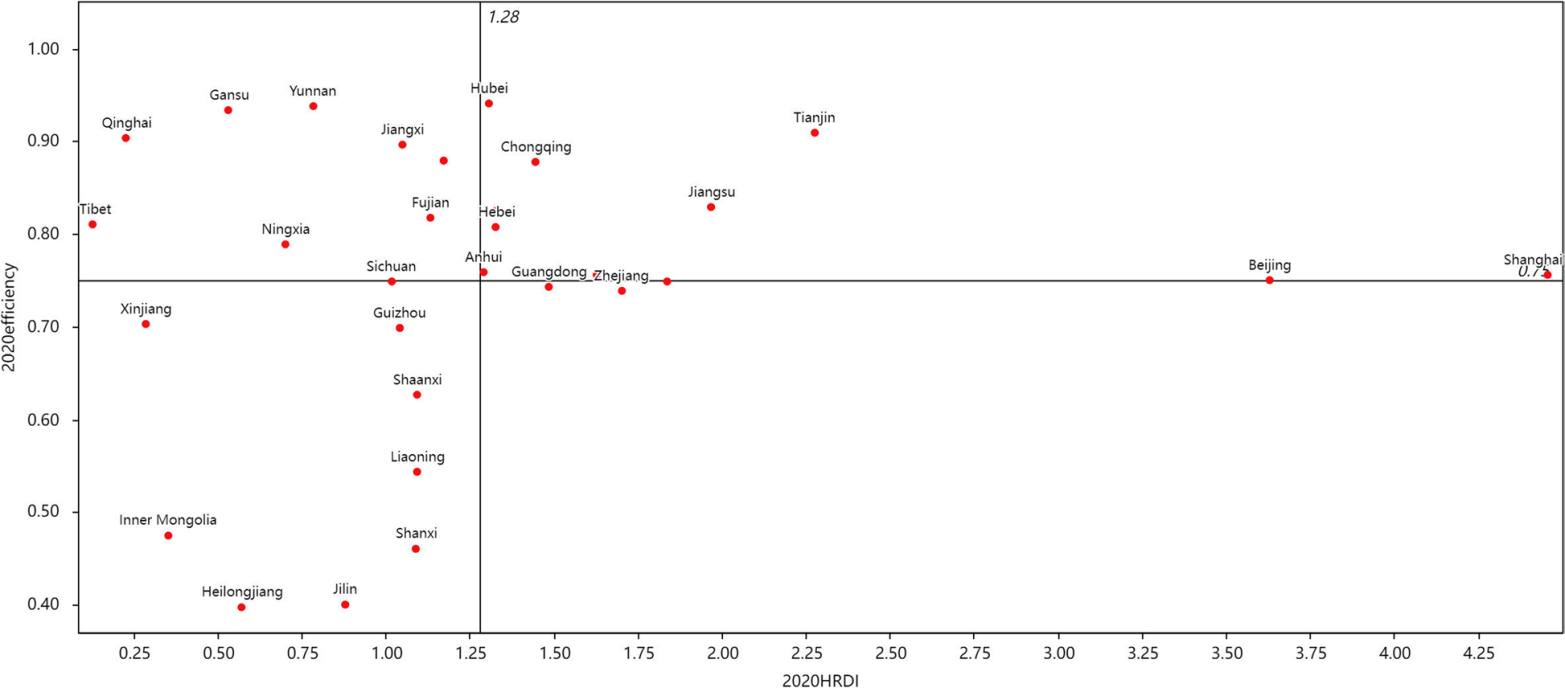
The comprehensive distribution plot of efficiency and CHRDI in 2020

Figures 2 and 3 only shows the comprehensive distribution plots of efficiency and CHRDI in 2016 and 2020. Approximately 2/3 of China’s provinces from 2016 and 2020 were located in the first and third quadrants, with provinces in the second and fourth quadrants near the axis. As shown in Figs. 2 and 3, 20 provinces were located in the first and third quadrants in 2016, and 19 provinces were located in the first and third quadrants in 2020, indicating that there may be a positive relationship between the level of resources allocation and efficiency in the PHC system. Jilin, Liaoning, and Heilongjiang had been in the third quadrant for five consecutive years, indicating low health resource allocation and low technical efficiency of the PHC system in the Northeast.

### The Moran’s index of the efficiency in the PHC system

The global Moran’s I of the efficiency in the PHC system from 2016 to 2020 was greater than 0, and the *P* values were all less than 0.05, indicating that the efficiency of the PHC system had a significant positive spatial correlation among all regions. The high-efficiency areas clustered with the high-efficiency areas, and the low-efficiency areas clustered with the low-efficiency areas. The global Moran’s I of the efficiency from 2016 to 2020 showed an increasing trend, indicating the spatial correlation of the efficiency of the PHC system between regions has been enhanced (Table 5).

**Table 5:** The global Moran’s I of the efficiency in PHC system, 2016–2020

I	E(I)	sd(I)	z	*p*
0.057	-0.033	0.034	2.659	0.004
0.050	-0.033	0.034	2.440	0.007
0.103	-0.033	0.034	3.983	< 0.001
0.101	-0.033	0.034	3.965	< 0.001
0.125	-0.033	0.034	4.601	< 0.001

“I” is the global Moran’s index

We further explored the local auto-correlation between 31 provinces and their neighboring locations. From 2018 to 2020, Guangxi and Yunnan were located in the high-high cluster areas, indicating that the efficiency of the PHC system in this region and its surrounding areas was highly efficient. From 2016 to 2020, Liaoning, Jilin, and Heilongjiang were located in the low-low cluster areas, indicating that the efficiency of the PHC system in the region and its surrounding areas was low. From 2017 to 2019, Shanxi was located in low–high cluster areas which suggested that the efficiency of the local PHC system was low, but the efficiency of the surrounding areas was high. The local Moran’s I of other provinces and cities was not significant (Table 6).

**Table 6:** The local Moran’s I of efficiency in PHC system, 2016–2020

Year	HH	HL	LL	LH
2016			Liaoning, Jilin, Heilongjiang	
2017			Liaoning, Jilin, Heilongjiang	Shanxi
2018	Guangxi, Yunnan		Liaoning, Jilin, Heilongjiang	Shanxi
2019	Guangxi, Yunnan		Liaoning, Jilin, Heilongjiang	Shanxi
2020	Guangxi, Yunnan		Liaoning, Jilin, Heilongjiang	

HH refers to a high-high cluster area. HL refers to a high-low cluster area, LL refers to a low-low cluster area, and LH refers to a low–high cluster area. Provinces with *P* values less than 0.05 are shown

### Analysis of factors affecting the efficiency based on the spatial Dubin model

The fixed SDM effect coefficient of the independent variable CHRDI was 0.912 and positively significant at the 1% level, indicating that the allocation of primary health resources had a significant positive contribution to the efficiency of the PHC system. The regression coefficients of population density, the proportion of government health expenditure in fiscal expenditure, and the proportion of service volume of the PHC system in health institutions were positive, but the results were not significant. In contrast, the regression coefficient of the per capita GDP and the urbanization rate were significantly negative, indicating that the improvement of the economic level and urbanization were not conducive to the improvement of efficiency of the regional PHC system.

To accurately judge the spatial spillover effect of each variable on the efficiency of the PHC system, the regression results of the spatial Dubin model were further decomposed, and the model parts were divided into direct effect, indirect effect (spatial spillover effect), and total effect [32]. The total, direct, and indirect effects of the CHRDI were all significantly positive, indicating that the rational allocation of health resources not only facilitated the improvement of the efficiency of the PHC system in the region but also had a positive impact on the regions adjacent to it. The total, direct, and indirect effects of the urbanization rate were all significantly negative, indicating that urbanization in the region simultaneously hindered the efficiency of the PHC system in the surrounding areas and generated negative spatial spillover effects (Table 7).

**Table 7:** Test results of the spatial Dubin model

	Fixed effect SDM	Fixed effect SDM decomposition		
		Direct effect	Indirect effect	Total effect
CHRDI	0.912^a^	0.820^a^	1.471^a^	2.291^a^
Population density	0.371	0.325	-0.750	-1.075
per capita GDP	-0.043^b^	-0.048	-0.038	-0.009
Urbanization rate	-0.765^a^	-0.797^a^	-0.444^a^	-0.353^a^
Proportion of the elderly	-0.010	-0.046	0.567	0.521
Proportion of government health expenditure in fiscal expenditure	0.152	0.077	1.083	1.160
The proportion of hospitals in health institutions	-0.013	-0.067	0.800	0.731
Proportion of service volume of PHC system in health institutions	0.298	0.247	0.659	0.904
Wx CHRDI	2.376^a^			
Wx Population density	-1.164^b^			
Wx per capital GDP	-0.036			
Wx Urbanization rate	-0.206^a^			
Wx Proportion of the elderly	-0.729			
Wx Proportion of government health expenditure in fiscal expenditure	1.545			
Wx Proportion of hospitals in health institutions	1.060			
Wx Proportion of service volume of PHC system in health institutions	1.004			
R^2^	0.012			
Log-likelihood	108.601			
N	155			

Wx refers to the spatial lag term of the independent variable. CHRDI refers to the comprehensive health resource density index, and GDP refers to the Gross Domestic Product

^a^means significant at the 99% level

^b^means significant at the 90% level

## Discussion

The results of this study revealed that the allocation of PHC system resources in China gradually increased from 2016 to 2020, and the efficiency of the PHC system as a whole showed a decline. The spatial Dubin model confirmed that the allocation of PHC system resources had a positive effect on the efficiency of the PHC system. The reasonable allocation of health care resources not only contributed to the improvement of the efficiency of the local PHC system but also positively impacted the adjacent regions. The effect of the per capita GDP and the urbanization rate on the efficiency of the PHC system was negative.

The allocation standard of health resources cannot be separated from the population index and geographical area index, but to consider the allocation of health resources by a single index is biased. Zheng et al. pointed out that the health human resources per 1,000 of the population in certain regions were much higher than the national and provincial average levels, but in fact, there was an obvious shortage of medicine [39]. The health resources have to be transformed into health services before they can be utilized, and the utilization degree depends on the proximity and contact density of the population to the resources [40]. Therefore, it would not be a rigorous analysis to separate the discussion of demographic factors and geographical factors [41]. The HRDI used in this study considered the population and geographical area and considered the equity and accessibility of health care resource distribution, so the measured health resources allocation was closer to the actual situation. The results of this study show that the overall allocation of primary health resources had gradually improved, which indicated that China paid more attention to the differences between and within regions while improving its investment. The allocation of primary health resources in first-tier cities such as Beijing and Shanghai was better than that in other provinces, while the allocation of primary health resources in western regions (Xinjiang, Tibet, Qinghai, etc.) was lower, and the reasons for this may be related to factors of geographical and environmental characteristics [42]. First-tier cities have relatively flat terrain and high population density, so primary health resources are concentrated at a high allocation, while backward cities are mountainous and have large geographical areas, making resource allocation relatively low.

The technical efficiency of the PHC system after bias correction was lower than that before bias correction because bootstrap DEA took into account the effect of random errors. Without controlling for the effect of random factors, the accounting of efficiency by traditional DEA models would be significantly overestimated. Meanwhile, no province in our country achieves technical efficiency, and the average value fluctuated between 0.73 and 0.82. We believe that the result is more realistic. Compared to similar studies, the TE in this study is lower than 0.83 in Extremadura, Spain [43] and 0.86 in Nouna Health District [44], but compared to the 2012–2016 efficiency in Zhang et al.’s study, the TE in this study has improved significantly [13]. The main reason is that China’s new healthcare reform has encouraged and guided residents to seek medical treatment in PHC institutions through policies such as “graded treatment” and “health insurance reform” [45] while improving the medical technology of the PHC system through the introduction of talent and infrastructure investment. However, both before and after correction, the technical efficiency of China’s PHC system showed a downward trend from 2016 to 2020, which is the same as Leng et al.’s point of view [46]. Blindly increasing input will lead to an inverted U-shaped development of technical efficiency. When the input reaches saturation, the efficiency will decline with the redundancy of input and ineffective management. Attempts to reduce the waste of resources by scaling back or closing down some of the less-performing institutions proved inappropriate [47]. Therefore, it is especially important to improve management, promote and encourage innovation, create a good working environment, and reduce the loss of people and idle equipment in the PHC system [48]. We are also concerned about inefficiencies in 2019 and 2020, which we believe may be the impact of the COVID-19 pandemic on the efficiency of the healthcare system, an observation that has been demonstrated by several studies [49–51]. Due to the substantial additional workload as part of the epidemic control, the PHC system experienced a shutdown from their routine care on other health conditions, and access to the routine functions of the PHC system such as outpatient visits was significantly affected.

When exploring the factors that influence efficiency, most studies use the Tobit model. However, this study found that when the efficiency of the PHC system has significant spatial correlation characteristics, the coefficient estimated by non-spatial measurement methods will be biased or inconsistent. In this case, the spatial Dubin model can effectively consider the result deviation caused by the spatial spillover effect [52, 53]. The results of the spatial Dubin model showed that the allocation of resources affects the technical efficiency of the PHC system. The rational allocation of resources can not only improve the technical efficiency of the local area but can also promote the efficiency of neighboring areas. Allocation equity and efficiency develop dynamically and harmoniously, which has been confirmed by several studies [54, 55]. For the spatial spillover effect of resource allocation, the most likely reason is the existence of competitive behavior and imitation demonstration effect, neighboring regions may imitate the efficient resource allocation level, so that the efficiency of their PHC system will increase. The comprehensive distribution plot of efficiency and HRDI showed few areas in the fourth quadrant (high allocation, low efficiency), confirming the results of the study. For the region in the third quadrant (low allocation, low efficiency), the basic health resources were weak and poorly allocated, and the equity of residents’ medical treatment may be poor [56]. The region in the second quadrant (low allocation, high efficiency) reflected the positive effect of the spatial spillover effect. In addition, the per capita GDP and the urbanization rate significantly affected the efficiency of the PHC system at the spatial level, probably because the growth of the economic level and deepening urbanization drive the government’s supply of health care resources in cities. At the same time, patients’ choice of medical treatment is less affected by objective conditions such as geographical distance, so they may choose more large hospitals for medical treatment, which hurts the PHC system [34]. Although the effect of aging on efficiency was not significant, the negative regression coefficient is still worthy of attention. With the growth of age and the deterioration of physical functions, elderly individuals have a higher demand for medical care [57]. However, at present, the elderly are more inclined to go to large hospitals for medical treatment and go to PHC institutions for prescriptions, which does not promote the efficiency of PHC institutions. Therefore, to improve technical efficiency, the external environmental factors of the region should be considered comprehensively, and health policies should be formulated according to the social and economic level of the city and the residents’ demand for affordability, accessibility, and quality in terms of medical treatment [58].

### Limitations

There were still some limitations in this study. First, although bootstrap-DEA was used in this study to obtain the technical efficiency after deviation correction, it was not further decomposed into pure technical efficiency and scale efficiency to explore more specific reasons for the insufficient efficiency. Second, when analyzing the allocation of health resources according to the population dimension, this study only considered the permanent population as the target population, which may affect the reflection of the real situation due to the existence of the floating population [59]. Finally, in the selection of independent variables of the spatial Dubin model, some unattainable and unquantifiable indicators, such as the degree of policy implementation, institutional implementation ability, and the level of institutional wages, had not been included in the influencing factors. Future research could measure the degree of government policy implementation and government implementation capacity to explore their impact on the efficiency of the PHC system.

## Conclusion

This study comprehensively analyzed the resource allocation level and efficiency of the PHC system in recent years, followed by an exploration of the impact of the level of resource allocation on efficiency. The position of the PHC system in the medical system has received increasing attention, and its health resource allocation has gradually improved. The allocation of primary health resources in large cities was higher than that in other cities. However, the efficiency of the primary health care system had regressed, and improving efficiency by blindly increasing the investment in medical resources had failed. Therefore, strengthening the management level of the PHC system is a feasible way to improve efficiency. The spatial Dubin model confirmed that the rational allocation of health resources not only contributes to the improvement of the efficiency of the local PHC system but also has a positive impact on neighboring regions. Therefore, this study proposes the following suggestions: Pay attention to the spatial spillover effect of the level of resource allocation and formulate differentiated measures for different regions. For regions with a high allocation of PHC system resources, the efficiency of the PHC system in the region and surrounding areas should be improved by helping and driving the surrounding areas. For regions with a low level of health resources, the allocation level of local resources must first be improved, then actively break down regional monopolies and learn from the modes of resource allocation in surrounding high-efficiency areas. Meanwhile, to respond to the current epidemiologic transition and future epidemic outbreaks more effectively, the transformation of PHC system resources such as adopting telehealth technologies to maximize primary care delivery is needed to optimize utilization of health resource inputs.

### Supplementary Information

**Additional file 1: Table S1.** Variance Inflation Factor (VIF) test. **Table S2-1.** Local Moran’s I of efficiency values of PHC system, 2016. **Table S2-2.** Local Moran’s I of efficiency values of PHC system, 2017. **Table S2-3.** Local Moran's I of efficiency values of PHC system, 2018. **Table S2-4.** Local Moran's I of efficiency values of PHC system, 2019. **Table S2-5.** Local Moran's I of efficiency values of PHC system, 2020.

## Data Availability

The datasets used during the current study are available from the corresponding author upon reasonable request.

## Abbreviations

PHC: Primary health care
HRDI: Health resource density index
CHRDI: Comprehensive health resource density index
TE: Technical efficiency
HH: High-high
LH: Low–high
LL: Low-low
HL: High-low
SLM: Spatial Lag model
SEM: Spatial Error model
SDM: Spatial Dubin model
