# Supplementary material for: Can the allocation of primary health care system resources affect efficiency? A spatial Dubin model study in China
Source: BMC Prim Care. 2024 Feb 21;25:62. doi: 10.1186/s12875-024-02290-y (PMC10882762; doi:10.1186/s12875-024-02290-y)
Supplement: Supplementary file 1 — Additional file 1: Table S1. Variance Inflation Factor (VIF) test. Table S2-1. Local Moran’s I of efficiency values of PHC system, 2016. Table S2-2. Local Moran’s I of efficiency values of PHC system, 2017. Table S2-3. Local Moran's I of efficiency values of PHC system, 2018. Table S2-4. Local Moran's I of efficiency values of PHC system, 2019. Table S2-5. Local Moran's I of efficiency values of PHC system, 2020. [file 12875_2024_2290_MOESM1_ESM.docx]

**Supplementary material 1 to the manuscript ”Can the allocation of primary health care system resources affect efficiency? A spatial Dubin model study in China”**

**Variance Inflation Factor (VIF) test**

The variance inflation factor test was performed on the independent and control variables included in the spatial Dubin model, when 0<VIF<10, there is no multi-collinearity.

**Table S1** Variance Inflation Factor (VIF) test

|  | VIF | 1/VIF |
| --- | --- | --- |
| X1 | 5.49 | 0.18 |
| X4 | 5.75 | 0.17 |
| X2 | 5.54 | 0.18 |
| X8 | 4.11 | 0.24 |
| X7 | 2.44 | 0.41 |
| X3 | 1.80 | 0.55 |
| X5 | 1.46 | 0.68 |
| X6 | 1.14 | 0.87 |

**Supplementary material 2 to the manuscript ”Can the allocation of primary health care system resources affect efficiency? A spatial Dubin model study in China”**

**The local Moran's I of the efficiency of the PHC system, 2016-2020**

From 2016 to 2020, the local Moran’s I was significant in Liaoning, Jilin and Heilongjiang. From 2017 to 2019, the local Moran’s I of Shanxi was significant; From 2018 to 2020, the local Moran’s I was significant in Guangxi and Yunnan. (Table S2-1 to Table S2-5)

**Table S2-1** Local Moran's I of efficiency values of PHC system, 2016

|  | Ii | E(Ii) | sd(Ii) | z | *p* |
| --- | --- | --- | --- | --- | --- |
| Beijing | 0.001 | -0.033 | 0.233 | 0.149 | 0.441 |
| Tianjing | -0.155 | -0.033 | 0.232 | -0.526 | 0.299 |
| Hebei | -0.207 | -0.033 | 0.160 | -1.085 | 0.139 |
| Shanxi | -0.248 | -0.033 | 0.147 | -1.466 | 0.071 |
| Inner Mongolia | 0.084 | -0.033 | 0.123 | 0.953 | 0.170 |
| **Liaoning** | **0.536** | **-0.033** | **0.144** | **3.950** | **＜0.001** |
| **Jilin** | **0.808** | **-0.033** | **0.196** | **4.299** | **＜0.001** |
| **Heilongjiang** | **0.543** | **-0.033** | **0.195** | **2.957** | **0.002** |
| Shanghai | 0.012 | -0.033 | 0.182 | 0.247 | 0.402 |
| Jiangsu | 0.085 | -0.033 | 0.180 | 0.656 | 0.256 |
| Zhejiang | 0.027 | -0.033 | 0.180 | 0.338 | 0.368 |
| Anhui | 0.117 | -0.033 | 0.172 | 0.870 | 0.192 |
| Fujian | -0.101 | -0.033 | 0.113 | -0.602 | 0.274 |
| Jiangxi | 0.090 | -0.033 | 0.133 | 0.925 | 0.177 |
| Shandong | 0.003 | -0.033 | 0.126 | 0.291 | 0.385 |
| Henan | -0.055 | -0.033 | 0.104 | -0.208 | 0.418 |
| Hubei | 0.064 | -0.033 | 0.126 | 0.768 | 0.221 |
| Hunan | 0.073 | -0.033 | 0.120 | 0.887 | 0.188 |
| Guangdong | 0.017 | -0.033 | 0.111 | 0.455 | 0.324 |
| Guangxi | 0.113 | -0.033 | 0.131 | 1.119 | 0.131 |
| Hainan | 0.102 | -0.033 | 0.138 | 0.982 | 0.163 |
| Chongqing | 0.026 | -0.033 | 0.125 | 0.478 | 0.316 |
| Sichuan | 0.001 | -0.033 | 0.128 | 0.270 | 0.394 |
| Guizhou | -0.161 | -0.033 | 0.119 | -1.072 | 0.142 |
| Yunnan | 0.068 | -0.033 | 0.114 | 0.890 | 0.187 |
| Xizang | 0.042 | -0.033 | 0.079 | 0.961 | 0.168 |
| Shaanxi | -0.078 | -0.033 | 0.094 | -0.479 | 0.316 |
| Gansu | -0.036 | -0.033 | 0.166 | -0.017 | 0.493 |
| Qinghai | 0.010 | -0.033 | 0.175 | 0.249 | 0.402 |
| Ningxia | -0.015 | -0.033 | 0.116 | 0.163 | 0.435 |
| Xinjiang | -0.006 | -0.033 | 0.073 | 0.373 | 0.355 |

Note: Ii is the local Moran’s index

**Table S2-2** Local Moran's I of efficiency values of PHC system, 2017

|  | Ii | E(Ii) | sd(Ii) | z | *p* |
| --- | --- | --- | --- | --- | --- |
| Beijing | 0.016 | -0.033 | 0.233 | 0.212 | 0.416 |
| Tianjing | -0.082 | -0.033 | 0.232 | -0.211 | 0.416 |
| Hebei | -0.282 | -0.033 | 0.160 | -1.555 | 0.060 |
| **Shanxi** | **-0.404** | **-0.033** | **0.146** | **-2.536** | **0.006** |
| Inner Mongolia | 0.035 | -0.033 | 0.123 | 0.553 | 0.290 |
| **Liaoning** | **0.475** | **-0.033** | **0.144** | **3.531** | **＜0.001** |
| **Jilin** | **0.774** | **-0.033** | **0.196** | **4.125** | **＜0.001** |
| **Heilongjiang** | **0.577** | **-0.033** | **0.195** | **3.129** | **0.001** |
| Shanghai | 0.010 | -0.033 | 0.182 | 0.241 | 0.405 |
| Jiangsu | 0.024 | -0.033 | 0.180 | 0.320 | 0.374 |
| Zhejiang | 0.010 | -0.033 | 0.180 | 0.238 | 0.406 |
| Anhui | 0.070 | -0.033 | 0.172 | 0.601 | 0.274 |
| Fujian | -0.138 | -0.033 | 0.112 | -0.937 | 0.174 |
| Jiangxi | 0.075 | -0.033 | 0.133 | 0.818 | 0.207 |
| Shandong | 0.006 | -0.033 | 0.125 | 0.317 | 0.376 |
| Henan | -0.035 | -0.033 | 0.104 | -0.015 | 0.494 |
| Hubei | 0.111 | -0.033 | 0.126 | 1.145 | 0.126 |
| Hunan | 0.110 | -0.033 | 0.119 | 1.200 | 0.115 |
| Guangdong | 0.017 | -0.033 | 0.111 | 0.454 | 0.325 |
| Guangxi | 0.120 | -0.033 | 0.130 | 1.179 | 0.119 |
| Hainan | 0.088 | -0.033 | 0.138 | 0.879 | 0.190 |
| Chongqing | 0.043 | -0.033 | 0.125 | 0.613 | 0.270 |
| Sichuan | 0.012 | -0.033 | 0.127 | 0.357 | 0.361 |
| Guizhou | -0.210 | -0.033 | 0.119 | -1.490 | 0.068 |
| Yunnan | 0.079 | -0.033 | 0.113 | 0.991 | 0.161 |
| Xizang | 0.053 | -0.033 | 0.078 | 1.110 | 0.134 |
| Shaanxi | -0.048 | -0.033 | 0.093 | -0.16 | 0.437 |
| Gansu | 0.013 | -0.033 | 0.166 | 0.277 | 0.391 |
| Qinghai | 0.036 | -0.033 | 0.175 | 0.399 | 0.345 |
| Ningxia | -0.015 | -0.033 | 0.115 | 0.160 | 0.436 |
| Xinjiang | 0.001 | -0.033 | 0.072 | 0.478 | 0.316 |

Note: Ii is the local Moran’s index

**Table S2-3** Local Moran's I of efficiency values of PHC system, 2018

|  | Ii | E(Ii) | sd(Ii) | z | *p* |
| --- | --- | --- | --- | --- | --- |
| Beijing | 0.006 | -0.033 | 0.234 | 0.168 | 0.433 |
| Tianjing | -0.041 | -0.033 | 0.232 | -0.031 | 0.487 |
| Hebei | -0.282 | -0.033 | 0.160 | -1.556 | 0.060 |
| **Shanxi** | **-0.385** | **-0.033** | **0.146** | **-2.408** | **0.008** |
| Inner Mongolia | -0.037 | -0.033 | 0.122 | -0.033 | 0.487 |
| **Liaoning** | **0.496** | **-0.033** | **0.143** | **3.688** | **＜0.001** |
| **Jilin** | **1.181** | **-0.033** | **0.196** | **6.198** | **＜0.001** |
| **Heilongjiang** | **1.176** | **-0.033** | **0.195** | **6.192** | **＜0.001** |
| Shanghai | 0.012 | -0.033 | 0.182 | 0.249 | 0.402 |
| Jiangsu | 0.051 | -0.033 | 0.180 | 0.471 | 0.319 |
| Zhejiang | 0.027 | -0.033 | 0.180 | 0.334 | 0.369 |
| Anhui | 0.097 | -0.033 | 0.172 | 0.758 | 0.224 |
| Fujian | -0.108 | -0.033 | 0.111 | -0.671 | 0.251 |
| Jiangxi | 0.103 | -0.033 | 0.132 | 1.035 | 0.150 |
| Shandong | 0.011 | -0.033 | 0.125 | 0.352 | 0.362 |
| Henan | 0.003 | -0.033 | 0.103 | 0.350 | 0.363 |
| Hubei | 0.145 | -0.033 | 0.125 | 1.426 | 0.077 |
| Hunan | 0.110 | -0.033 | 0.118 | 1.207 | 0.114 |
| Guangdong | 0.038 | -0.033 | 0.110 | 0.645 | 0.260 |
| **Guangxi** | **0.207** | **-0.033** | **0.130** | **1.854** | **0.032** |
| Hainan | 0.107 | -0.033 | 0.137 | 1.021 | 0.154 |
| Chongqing | 0.100 | -0.033 | 0.124 | 1.079 | 0.140 |
| Sichuan | 0.047 | -0.033 | 0.127 | 0.635 | 0.263 |
| Guizhou | -0.047 | -0.033 | 0.118 | -0.116 | 0.454 |
| **Yunnan** | **0.159** | **-0.033** | **0.113** | **1.713** | **0.043** |
| Xizang | 0.034 | -0.033 | 0.076 | 0.884 | 0.188 |
| Shaanxi | -0.078 | -0.033 | 0.092 | -0.490 | 0.312 |
| Gansu | 0.044 | -0.033 | 0.166 | 0.466 | 0.321 |
| Qinghai | 0.040 | -0.033 | 0.175 | 0.421 | 0.337 |
| Ningxia | -0.007 | -0.033 | 0.114 | 0.231 | 0.409 |
| Xinjiang | -0.025 | -0.033 | 0.070 | 0.113 | 0.455 |

Note: Ii is the local Moran’s index

**Table S2-4** Local Moran's I of efficiency values of PHC system, 2019

| 2019 | Ii | E(Ii) | sd(Ii) | z | *p* |
| --- | --- | --- | --- | --- | --- |
| Beijing | ＜0.001 | -0.033 | 0.233 | 0.142 | 0.443 |
| Tianjing | -0.050 | -0.033 | 0.232 | -0.073 | 0.471 |
| Hebei | -0.226 | -0.033 | 0.160 | -1.200 | 0.115 |
| **Shanxi** | **-0.299** | **-0.033** | **0.146** | **-1.819** | **0.034** |
| Neimenggu | 0.006 | -0.033 | 0.123 | 0.318 | 0.375 |
| **Liaoning** | **0.446** | **-0.033** | **0.144** | **3.334** | **＜0.001** |
| **Jilin** | **1.094** | **-0.033** | **0.196** | **5.757** | **＜0.001** |
| **Heilongjiang** | **1.108** | **-0.033** | **0.195** | **5.850** | **＜0.001** |
| Shanghai | -0.002 | -0.033 | 0.182 | 0.171 | 0.432 |
| Jiangsu | 0.027 | -0.033 | 0.180 | 0.334 | 0.369 |
| Zhejiang | 0.003 | -0.033 | 0.180 | 0.201 | 0.420 |
| Anhui | 0.008 | -0.033 | 0.172 | 0.240 | 0.405 |
| Fujian | -0.003 | -0.033 | 0.112 | 0.027 | 0.489 |
| Jiangxi | 0.101 | -0.033 | 0.133 | 1.016 | 0.155 |
| Shandong | -0.019 | -0.033 | 0.125 | 0.113 | 0.455 |
| Henan | -0.018 | -0.033 | 0.104 | 0.150 | 0.440 |
| Hubei | 0.122 | -0.033 | 0.126 | 1.233 | 0.109 |
| Hunan | 0.078 | -0.033 | 0.119 | 0.934 | 0.175 |
| Guangdong | 0.012 | -0.033 | 0.111 | 0.411 | 0.341 |
| **Guangxi** | **0.185** | **-0.033** | **0.130** | **1.676** | **0.047** |
| Hainan | 0.109 | -0.033 | 0.138 | 1.032 | 0.151 |
| Chongqing | 0.107 | -0.033 | 0.125 | 1.130 | 0.129 |
| Sichuan | 0.021 | -0.033 | 0.127 | 0.431 | 0.333 |
| Guizhou | 0.045 | -0.033 | 0.119 | 0.658 | 0.255 |
| **Yunnan** | **0.184** | **-0.033** | **0.113** | **1.916** | **0.028** |
| Xizang | 0.014 | -0.033 | 0.078 | 0.615 | 0.269 |
| Shannxi | -0.063 | -0.033 | 0.093 | -0.323 | 0.373 |
| Gansu | 0.088 | -0.033 | 0.166 | 0.731 | 0.232 |
| Qinghai | 0.109 | -0.033 | 0.175 | 0.817 | 0.207 |
| Ningxia | -0.008 | -0.033 | 0.115 | 0.224 | 0.411 |
| Xinjiang | -0.005 | -0.033 | 0.072 | 0.396 | 0.346 |

Note: Ii is the local Moran’s index

**Table S2-5** Local Moran's I of efficiency values of PHC system, 2020

|  | Ii | E(Ii) | sd(Ii) | z | *p* |
| --- | --- | --- | --- | --- | --- |
| Beijing | -0.001 | -0.033 | 0.234 | 0.138 | 0.445 |
| Tianjing | -0.191 | -0.033 | 0.233 | -0.676 | 0.249 |
| Hebei | -0.087 | -0.033 | 0.160 | -0.339 | 0.367 |
| Shanxi | -0.048 | -0.033 | 0.145 | -0.102 | 0.459 |
| Inner Mongolia | 0.095 | -0.033 | 0.121 | 1.060 | 0.145 |
| **Liaoning** | **0.533** | **-0.033** | **0.143** | **3.965** | **＜0.001** |
| **Jilin** | **1.189** | **-0.033** | **0.196** | **6.231** | **＜0.001** |
| **Heilongjiang** | **1.179** | **-0.033** | **0.195** | **6.203** | **＜0.001** |
| Shanghai | 0.001 | -0.033 | 0.182 | 0.186 | 0.426 |
| Jiangsu | 0.026 | -0.033 | 0.180 | 0.330 | 0.371 |
| Zhejiang | -0.012 | -0.033 | 0.180 | 0.117 | 0.454 |
| Anhui | 0.006 | -0.033 | 0.172 | 0.230 | 0.409 |
| Fujian | 0.059 | -0.033 | 0.110 | 0.840 | 0.200 |
| Jiangxi | 0.171 | -0.033 | 0.131 | 1.553 | 0.060 |
| Shandong | 0.001 | -0.033 | 0.124 | 0.275 | 0.392 |
| Henan | ＜0.001 | -0.033 | 0.101 | 0.327 | 0.372 |
| Hubei | 0.146 | -0.033 | 0.124 | 1.444 | 0.074 |
| Hunan | 0.108 | -0.033 | 0.117 | 1.201 | 0.115 |
| Guangdong | -0.019 | -0.033 | 0.109 | 0.135 | 0.446 |
| **Guangxi** | **0.199** | **-0.033** | **0.129** | **1.806** | **0.035** |
| Hainan | 0.176 | -0.033 | 0.136 | 1.532 | 0.063 |
| Chongqing | 0.079 | -0.033 | 0.123 | 0.917 | 0.180 |
| Sichuan | -0.009 | -0.033 | 0.126 | 0.196 | 0.422 |
| Guizhou | -0.109 | -0.033 | 0.117 | -0.642 | 0.260 |
| **Yunnan** | **0.158** | **-0.033** | **0.111** | **1.718** | **0.043** |
| Xizang | 0.044 | -0.033 | 0.074 | 1.047 | 0.148 |
| Shaanxi | -0.087 | -0.033 | 0.090 | -0.597 | 0.275 |
| Gansu | 0.119 | -0.033 | 0.165 | 0.920 | 0.179 |
| Qinghai | 0.164 | -0.033 | 0.175 | 1.129 | 0.129 |
| Ningxia | -0.002 | -0.033 | 0.113 | 0.277 | 0.391 |
| Xinjiang | -0.016 | -0.033 | 0.067 | 0.264 | 0.396 |
